# Exploring the pharmacological action mechanism of *Ligusticum Chuanxiong*-*Piper Longum* couplet medicines on the treatment of migraine based on network pharmacology

**DOI:** 10.3389/fphar.2022.923188

**Published:** 2022-09-27

**Authors:** Ti Li, Shupeng Guo, Meixi Lu, Fang Lu, Tianjiao Lu, Chunze Zheng

**Affiliations:** ^1^ Department of Encephalopathy, Harbin Traditional Chinese Medicine Hospital, Harbin, China; ^2^ Department of Pharmacy, Harbin Traditional Chinese Medicine Hospital, Harbin, China; ^3^ Department of Pharmacology, School of Traditional Chinese Medicine, Beijing University of Chinese Medicine, Beijing, China; ^4^ National Medical Master Lu Fang Inheritance Studio, Harbin Traditional Chinese Medicine Hospital, Harbin, China; ^5^ Department of Acupuncture and Moxibustion, Harbin Traditional Chinese Medicine Hospital, Harbin, China

**Keywords:** Ligusticum chuanxiong Hort.-Piper longum L., migraine, network pharmacology, mechanism, neuroactive ligand-receptor interaction pathway

## Abstract

**Objective:** To study the mechanisms of the *Ligusticum chuanxiong* Hort.–*Piper longum* L. herbal pair (LPHP) in the treatment of migraine using network pharmacology.

**Methods:** The active constituents of LPHP and their targets were searched for and screened using the Chinese Medicine System Pharmacology Database. Genes related to migraine were searched on GeneCards, Online Mendelian Inheritance in Man and other databases. Cytoscape was used to construct and combine active component–target and disease–target networks. The core target was screened by network topology analysis, and the Metascape database was used for gene ontology analysis of key targets and Kyoto Encyclopedia of Genes and Genomes pathway enrichment analysis to explore the molecular mechanisms in the treatment of migraine.

**Results:** A total of 28 active constituents of LPHP were obtained through database screening and literature review, and 60 cross-linking targets were obtained. The target sites were analysed using a protein–protein interaction network to obtain six target proteins with a greater degree of relevance. These were identified as the main targets for the treatment of hypertension, and these key targets were found to be associated with 20 signalling pathways, including neuroactive ligand–receptor interaction, the calcium signalling pathway, the cGMP–PKG signalling pathway, pathways in cancer and the cyclic adenosine 3′,5′-cyclic monophosphate (cAMP) signalling pathway.

**Conclusion:** This study reveals the molecular mechanism of LPHP in the treatment of migraine from the perspective of network pharmacology and provides a basis for further research and molecular mechanism research.

## Introduction

Migraine is a common and frequently occurring chronic neurovascular disease. Its onset is characterised by repeated attacks (Tan and Fan, 2012), seriously endangering the physical and mental health of patients. It has been listed by the World Health Organization as a chronic disease that seriously affects patients’ quality of life.

In clinical application, *Ligusticum chuanxiong–Piper longum* (*Lc–Pl*) couplet medicines have been shown to have a good effect on migraine. For example, a previous study by our research group (Li et al., 2019) demonstrated a better cure rate of headaches in the treatment group than in the control group, and there were no adverse reactions during follow-up. However, there are relatively few studies on the mechanism of *Lc–Pl* couplet medicines in the treatment of migraine. The material system of these medicines is complex, as is the network relationship between the effective material basis and the target for the treatment of migraine. Therefore, this paper discusses the mechanism of *Lc–Pl* couplet medicines in the treatment of migraine based on a network pharmacology approach.

Network pharmacology is a discipline based on the theories of systems biology and multidirectional pharmacology, using omics and network visualisation technology to reveal the complex biological network relationships among drugs, genes, diseases and targets, and to predict the pharmacological action mechanisms of drugs (Hopkins, 2008a). The network pharmacology approach has been widely used in traditional Chinese medicine target research and has important guiding significance for clarifying its pharmacological mechanisms (Li and Su, 2016).

**FIGURE 1:**
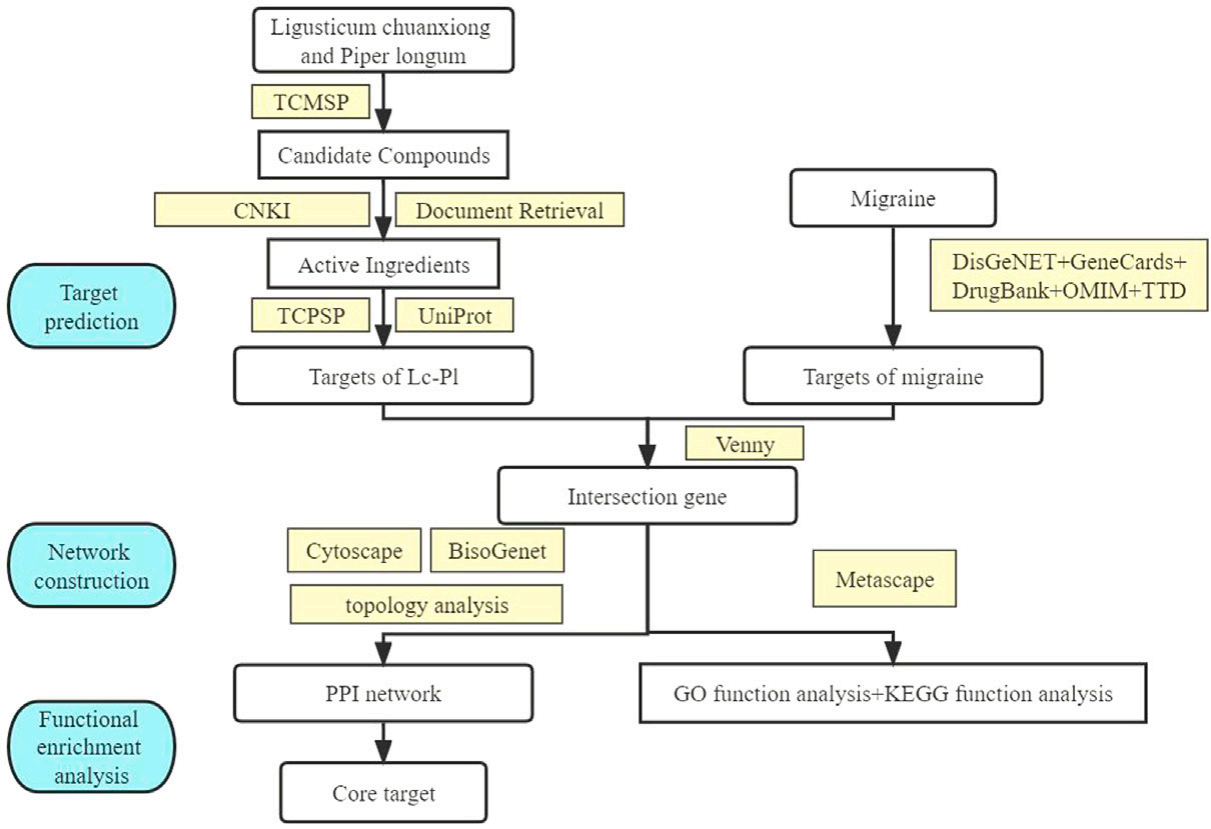
A flowchart of the study approach.

In this study, we investigated the blood inflow and active ingredients of *Lc–Pl* couplet medicines using a network pharmacology framework to reveal the action mechanisms of these medicines from a multi-component, multi-target and multi-channel perspective. A flowchart of the study approach is shown in Figure 1.

## Materials and methods

### Collection of chemical components in *Ligusticum chuanxiong–Piper longum* couplet medicines

This research was based on the traditional Chinese medicine systems pharmacology database and analysis platform (TCMSP, http://tcmspw.com/tcmsp.php). On this platform, we searched for the chemical components of *Ligusticum chuanxiong* and *Piper longum*, using these Latin taxonomic names as keywords.

### Screening of active chemical components and collection of corresponding targets

The collected chemical components were screened according to the two following conditions: oral bioavailability (OB) ≥ 30% and drug-likeness (DL) ≥ 0.18. For the common active compounds that did not meet the screening criteria, the relevant literature was searched for supplements through the China National Knowledge Infrastructure (CNKI) platform using “*Ligusticum chuanxiong*“ and “*Piper longum*” as keywords. Finally, chemical components with high activity were selected as candidate chemical components, and potential targets corresponding to candidate chemical components were collected on the TCMSP.

After screening, the UniProt database (https://www.uniprot.org) was used to standardise the protein target information of the compounds.

### Identification of disease targets

Disease-related targets for migraine were collected through the DisGeNET database (http://www.disgenet.org), the DrugBank database (http://www.drugbank.ca), the GeneCards database (https://www.genecards.org), the Online Mendelian Inheritance in Man database (OMIM, http://www.omim.org), and the Therapeutic Target Database (TTD, http://bidd.nus.edu.sg/group/cjttd).

### Construction of drug-component–target–disease network

The intersection of potential drug targets and disease-related targets was screened using the WeChat website (http://www.bioinformatics.com.cn/). The active ingredients of the drug and the screened intersection targets were uploaded to the Cytoscape 3.7.2 software to generate the drug-component–target network diagram, and the key chemical components and targets were obtained through network topology analysis.

### Screening of action targets and construction of protein–protein interaction network

The intersection target data were uploaded to the BisoGenet database to construct the protein–protein interaction (PPI) diagram. We then conducted a topology analysis of the PPI network and calculated the topology parameter values, including the degree of connectivity (Degree), betweenness, closeness, local average connectivity, and neighbourhood connectivity. The core targets with the strongest interactions were determined according to the degree value, which refers to the number of connections between a node and other nodes. A higher degree value thus reflects a wider set of connections between a given node and other nodes in the network.

### Gene ontology function enrichment analysis and pathway enrichment analysis based on the kyoto encyclopedia of genes and genomes

We conducted gene ontology (GO) function enrichment analysis and Kyoto Encyclopedia of Genes and Genomes (KEGG) pathway enrichment analysis on the intersection targets using the Metascape database, and screened the main action pathways of *Lc–Pl* couplet medicines in the treatment of migraine.

## Results

### Collection of chemical components in *Ligusticum chuanxiong–Piper longum* couplet medicines

Using TCMSP, all 189 chemical components of *Ligusticum chuanxiong* and 104 chemical components of *Piper longum* were obtained.

### Screening of active chemical components and related targets

The collected chemical components were screened using the conditions OB ≥ 30% and DL ≥ 0.18, and the active ingredients in the blood were screened out. From this process, six active components of *Ligusticum chuanxiong* (seven components in total, one of which has no effective target) and 13 active components of *Piper longum* (15 components in total, two of which have no effective target) were obtained. Then, common ingredients that did not meet the screening criteria were identified according to relevant reports published on CNKI, giving eight *Ligusticum chuanxiong* supplements (including ligustrazine) and one *Piper longum* supplement (piplartin).

The active ingredients of the *Lc–Pl* couplet medicines were summarised, resulting in no duplicate ingredients. A total of 28 chemical ingredients were obtained, which were classed as candidate ingredients for the *Lc–Pl* couplet medicines (Table 1). We then collected the corresponding gene targets of the candidate chemical ingredients on the TCMSP platform. The results returned 53 targets for *Ligusticum chuanxiong* and 82 for *Piper longum*, giving a total of 113 after deduplication. They were named after UniProt standardisation, and after a further deduplication process, a total of 110 potential targets of active ingredients were obtained.

**TABLE 1:** Basic information of the active ingredients of Ligusticum chuanxiong-Piper longum couplet medicines.

Source	MOL ID	Compound name	MW	OB/%	BBB	DL	Selection principle
Ligusticum chuanxiong	MOL001494	Mandenol	308.56	42	1.14	0.19	OB-DL principle
	MOL002135	Myricanone	356.45	40.6	−0.08	0.51	OB-DL principle
	MOL002140	Perlolyrine	264.3	65.95	0.15	0.27	OB-DL principle
	MOL002157	Wallichilide	412.57	42.31	0.73	0.71	OB-DL principle
	MOL000359	Sitosterol	414.79	36.91	0.87	0.75	OB-DL principle
	MOL000433	FA (Folic Acid)	441.45	68.96	−2.59	0.71	OB-DL principle
	MOL002202	Tetramethylpyrazine	136.22	20.01	1.05	0.03	Literature report
	MOL002208	Senkyunolide A	192.28	26.56	1.34	0.07	Literature report
	MOL002145	Senkyunolide-E	204.24	34.4	0.06	0.08	Literature report
	MOL004705	(3E,6S,7R)-3-butylidene-6,7-dihydroxy-4,5,6,7-tetrahydroisobenzofuran-1-one	224.28	34.34	−0.19	0.1	Literature report
	MOL002143	senkyunolide-C	204.24	46.8	0.5	0.08	Literature report
	MOL000360	FER(ferulic acid)	194.2	39.56	−0.03	0.06	Literature report
	MOL002201	Cis-ligustilide	190.26	51.3	1.24	0.07	Literature report
	MOL011782	Ligustilide	190.26	23.5	1.2	0.07	Literature report
Piper longum	MOL001555	ZINC03996196(Diaeudesmin)	386.48	52.35	0.08	0.62	OB-DL principle
	MOL001558	Sesamin	354.38	56.55	−0.08	0.83	OB-DL principle
	MOL001559	Piperlonguminine	273.36	30.71	0.27	0.18	OB-DL principle
	MOL001560	Pipernonaline	341.49	51.32	0.33	0.41	OB-DL principle
	MOL001561	Dehydropipernonaline	339.47	47.73	0.24	0.41	OB-DL principle
	MOL001586	N-(2,5-dimethoxyphenyl)-4-methoxybenzamide	287.34	60.7	0.33	0.18	OB-DL principle
	MOL001592	Piperine	285.37	42.52	0.62	0.23	OB-DL principle
	MOL001594	Pisatin	314.31	88.05	−0.22	0.64	OB-DL principle
	MOL001601	1,2,5,6-tetrahydrotanshinone	280.34	38.75	0.39	0.36	OB-DL principle
	MOL001607	ZINC03982454(beta-Sitosterol)	414.79	36.91	0.88	0.76	OB-DL principle
	MOL001610	Sylvatine	383.58	44	0.39	0.51	OB-DL principle
	MOL001614	(E,E,E)-11-(1,3-Benzodioxol-5-yl)-N-(2-methylpropyl)-2,4,10-undecatrienenamide (retrofractamide B)	353.5	42.72	0.34	0.43	OB-DL principle
	MOL001616	1-[1-oxo-9 (3,4-methylenedioxyphenyl)-2E,8E-nonadienyl] pyrrolidine (brachyamide B)	327.46	49.43	0.32	0.36	OB-DL principle
	MOL002848	Cis-Piplartine	317.37	96.65	0.37	0.24	Literature report

### Determination of disease targets

“Migraine” was used as the keyword to find the disease targets in the database, and UniProt was used to unify the target names into gene names. We obtained 481 targets from DisGeNET, 137 from DrugBank, 951 from GeneCards, 42 from OMIM, and 37 from TTD. The targets from the five disease databases were combined, and after removing duplicates, the set consisted of 1,236 disease targets.

### Construction of drug-component–target–disease network

**FIGURE 2:**
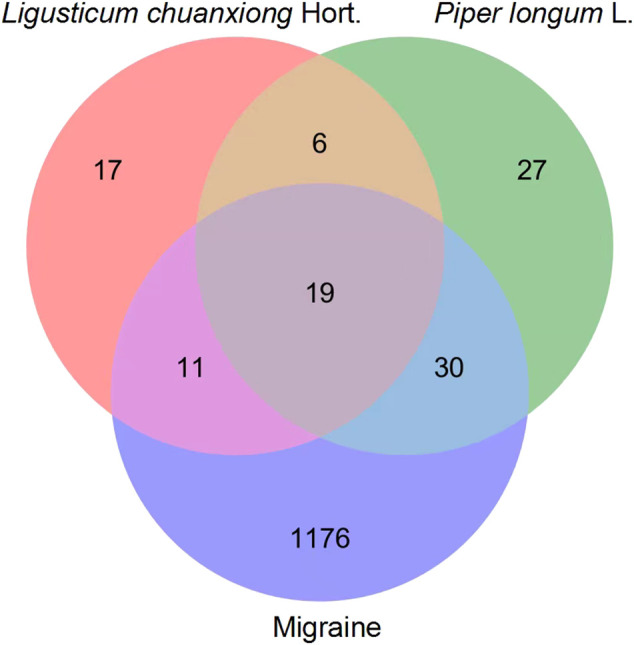
Venny diagram of active ingredient targets of Ligusticum chuanxiong-Piper longum couplet medicines and migraine targets.

A total of 60 intersections of potential targets of the *Lc–Pl* couplet medicines and disease-related targets of migraines were identified using the Venn diagram analysis tool on the Bioinformatics website (Figure 2), indicating 60 targets by which the active ingredients of *Lc–Pl* couplet medicines could directly act on migraine. The two drugs had a total of 19 common targets. In addition to the common targets, each had specific distinct targets (11 for *Ligusticum chuanxiong* and 30 for *Piper longum*), further indicating that there might be a synergistic relationship between these two traditional Chinese medicines. Overall, *Piper longum* had more disease targets; therefore, the order of contribution in the treatment of migraine was determined as *Piper longum* > *Ligusticum chuanxiong*.

**FIGURE 3:**
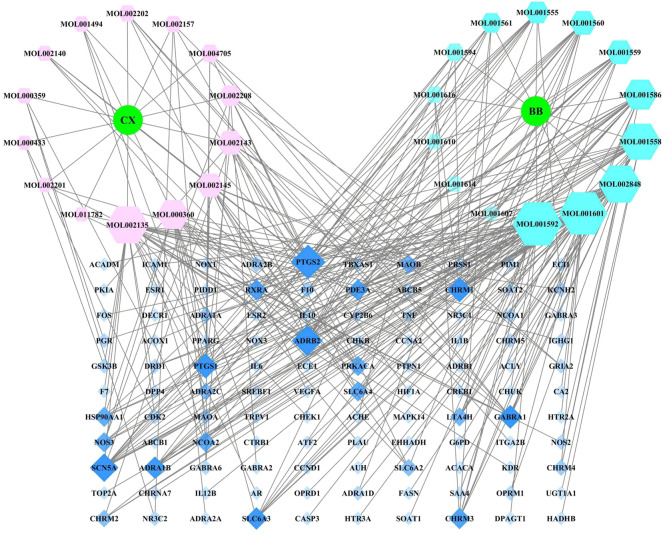
Network diagram of active ingredients of Ligusticum chuanxiong-Piper longum couplet medicines and migraine targets.

The 28 chemical components, the 60 targets, *Ligusticum chuanxiong* (CX) and *Piper longum* (BB) were used to construct an active ingredient–direct acting target network diagram in Cytoscape 3.7.2. The network diagram, as presented in Figure 3, contains 140 nodes (28 chemical component nodes, 110 target nodes, and 2 drug nodes) and 304 edges. The 28 chemical components were all related to diseases and all participated in the network construction.

In the network diagram, the hexagonal nodes of various colours represent the active ingredients of different drugs, and the squares represent targets; the area and colour transparency of each node represents its Degree value, with larger areas and darker shading representing increasing importance. As shown by the results, the *Lc–Pl* couplet medicines involve not only the phenomenon of one compound acting on multiple target proteins but also that of different compounds acting on the same target protein.

The network topology parameters for the treatment of migraine were analysed using the built-in Network Analyzer in CytoScape 3.7.2, and the Degree value of each node was calculated. The top-ranked chemical components for Degree were as follows: piperine (MOL001592), tetrahydro tanshinone I (MOL001601), myricetone (MOL002135), piplartin (MOL002848), sesamin (MOL001558), 4-methoxy-N-(2,5-dimethoxyphenyl) benzamide (MOL001586), ferulic acid (MOL000360), senkyunolide E (MOL002145), senkyunolide I (MOL002143), piperlonguminine (MOL001559), and pipernonaline (MOL001560).

The top-ranked targets for Degree were cyclooxygenase 2 (PTGS2/COX2), *ß* 2-adrenergic receptor (ADRB2), cardiac sodium channel gene (SCN5A), acetylcholine M1 receptor (CHRM1), cyclooxygenase 1 (PTGS1/COX1), γ-aminobutyric acid type A receptor α1 (GABRA1), α1 adrenergic receptor B (ADRA1B), solute carrier family 6 member 3 (SLC6A3), muscarinic acetylcholine receptor M3 (CHRM3), phosphodiesterase 3α (PDE3A), and retinoic acid X receptor *a* (RXRA). The above might thus be the key chemical components and action targets of *Lc–Pl* couplet medicines. These findings also reflect the multi-component and multi-target therapeutic characteristics of *Lc–Pl* couplet medicines on migraine.

### Screening of action targets and construction and analysis of protein–protein interaction network diagram

**FIGURE 4:**
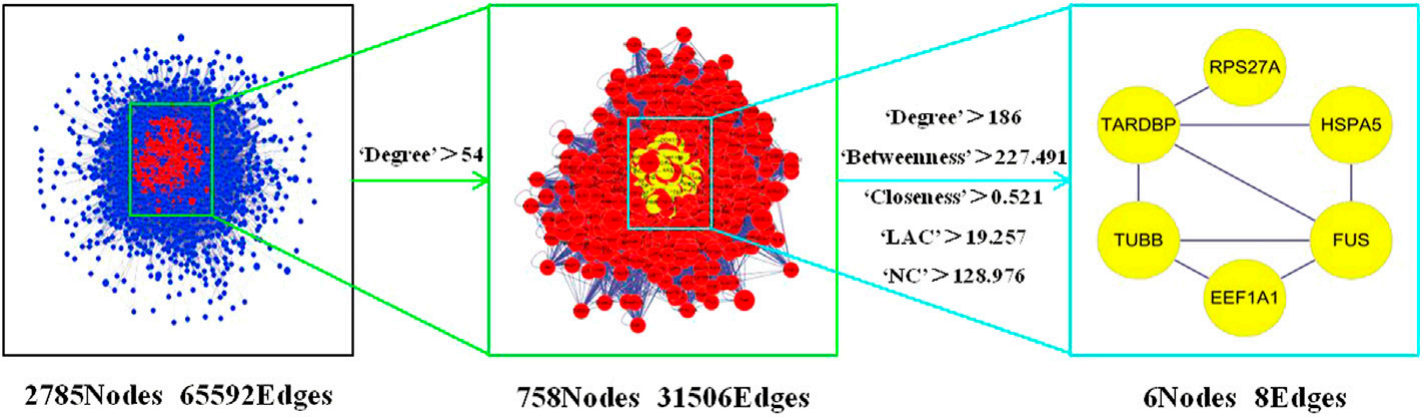
PPI network of target protein related to migraine and Ligusticum chuanxiong-Piper longum couplet medicines and flow chart of the screening target protein.

The 60 intersection targets were then uploaded to the BisoGenet plug-in of Cytoscape 3.7.2 to analyse the protein interactions between the targets, obtain their corresponding indirect action targets and construct the PPI network. The resulting network, containing 2,785 nodes and 65,592 edges, is shown in Figure 4. The network nodes represent target proteins, and the lines represent the relationships between the proteins.

In the PPI network diagram, the target proteins (core targets) with the strongest interaction values were screened under the conditions shown in Figure 4. The six top-ranked core targets by Degree (Table 2) were heat shock protein A5 (HSPA5), eukaryotic translation elongation factor 1A1 (EEF1A1), sarcoma fusion factor (FUS), ribosomal protein S27a (RPS27A), TAR-DNA binding protein (TARDBP), and β-tubulin (TUBB). These targets appear at the centre of the network diagram and represent the core targets of *Lc–Pl* couplet medicines for migraine, and these six core targets also affect each other. It is of interest to note that the closest relationship is indicated between TARDBP and FUS, which are both related to amyotrophic lateral sclerosis. (Lattante et al., 2013).

**TABLE 2:** Core target information of Ligusticum chuanxiong-Piper longum couplet medicines in the treatment of migraine.

No.	Gene names	Uniprot ID	Protein names	Degree	Betweenness	Closeness	LAC	NC
1	HSPA5	P11021	Endoplasmic reticulum chaperone BiP	268	3097.877559	0.576981707	44.34975369	133.1015038
2	EEF1A1	P68104	Elongation factor 1-alpha 1	228	2249.045008	0.568318318	46.36612022	139.261062
3	FUS	P35637	RNA-binding protein FUS	203	1746.475944	0.558259587	38.73584906	135.7910448
4	RPS27A	P62979	Ubiquitin-40S ribosomal protein S27a	198	2221.742055	0.555392517	35.86842105	130.0459184
5	TARDBP	Q13148	TAR DNA-binding protein 43	197	1327.309986	0.557027226	47.65838509	130.1230769
6	TUBB	P07437	Tubulin beta chain	188	1370.380358	0.553767374	33.14965986	147.8031915

### Gene ontology function enrichment analysis and kyoto encyclopedia of genes and genomes pathway enrichment analysis

**FIGURE 5:**
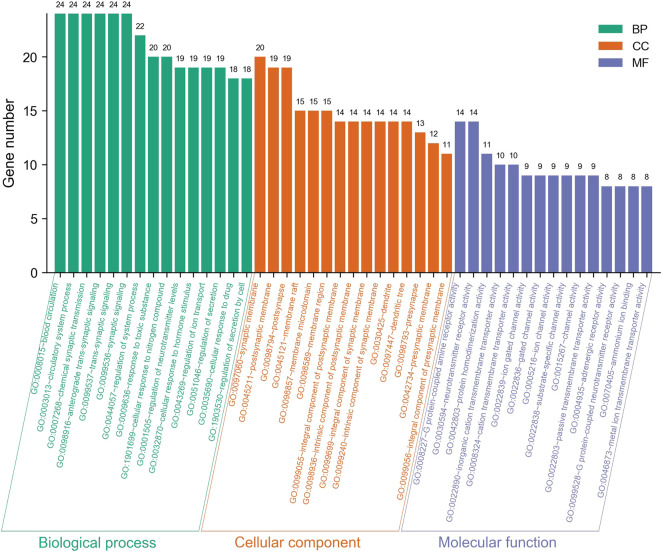
Histogram of GO enrichment analysis of Ligusticum chuanxiong-Piper longum couplet medicines in the treatment of migraine (top 15).

**FIGURE 6:**
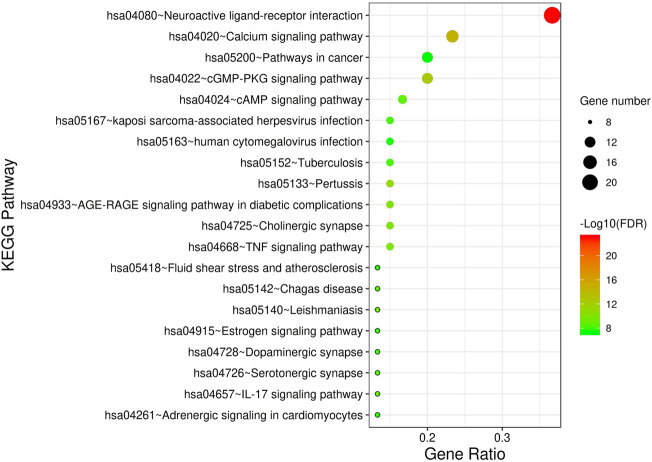
Bubble diagram of enrichment analysis of KEGG pathway of Ligusticum chuanxiong-Piper longum couplet medicines in the treatment of migraine (top 20).

The intersection targets were used for GO function enrichment analysis and KEGG pathway enrichment analysis in the Metascape database. Taking *p*-value as the screening criterion, the top 15 and the top 20 entries were selected for analysis, respectively, and the histogram and bubble chart were drawn using tools on the Bioinformatics website. The GO results are shown in Figure 5, and the KEGG results are shown in Figure 6.

As shown in Figure 5, the GO analysis results mainly focused on biological processes including those associated with blood circulation, circulatory system, chemical synaptic transmission, anterograde trans-synaptic signalling and trans-synaptic signalling, on cell components including the synaptic membrane and the postsynaptic membrane, and molecular functions including G protein-coupled amine receptor activity, neurotransmitter receptor activity and protein homodimerisation activity.

The KEGG pathway enrichment analysis screened 20 signal pathways, as shown in Figure 6. The size of each bubble in the figure represents the number of enriched genes in the relevant pathway, and the colour of each bubble represents the extent of enrichment of target genes in the pathway. The significant pathways (*p* < 0.01) include the neuroactive ligand–receptor interaction pathway, calcium signalling pathway, cGMP-PKG signalling pathway, pathways in cancer and the adenosine 3′,5′-cyclic monophosphate (cAMP) signalling pathway. On this basis, we infer that *Lc–Pl* couplet medicines might be the result of a multi-channel synergistic effect in the treatment of migraine.

**FIGURE 7:**
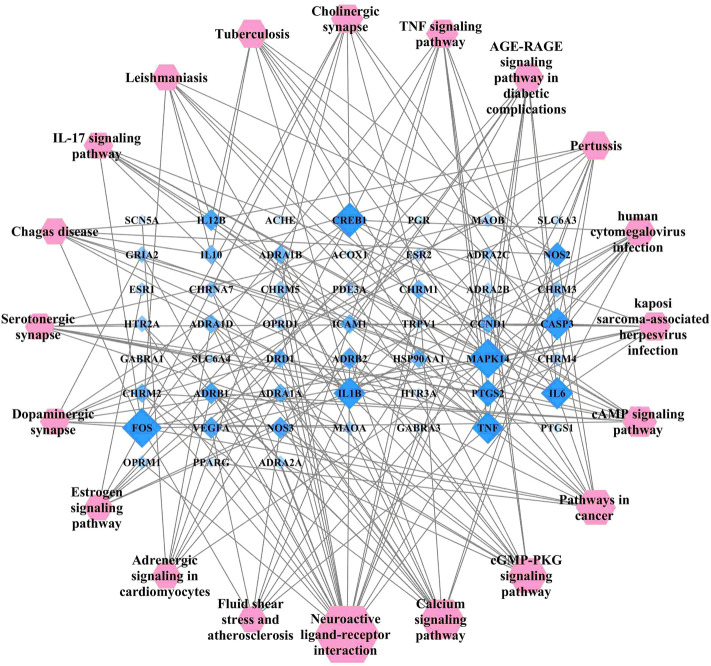
Network diagram of the KEGG pathway, the target of Ligusticum chuanxiong-Piper longum couplet medicines for the treatment of migraine.

The target pathway network for *Lc–Pl* couplet medicines, as presented in Figure 7, was constructed using Cytoscape 3.7.2. In this network, different targets are enriched by each signal pathway, indicating that the various active ingredients have different effects in terms of the regulation of targets and signal pathways. As shown in the figure, the most significant number of enriched genes was identified in the neural activity ligand–receptor interaction pathway, which might thus be an important signal pathway for *Lc–Pl* couplet medicines in the treatment of migraine.

**FIGURE 8:**
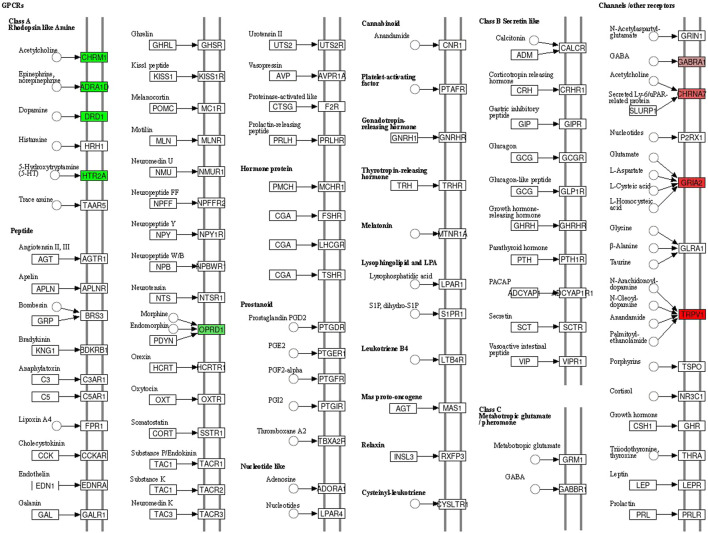
Specific action mechanism of enriched genes in neural activity ligand receptor interaction signal pathway.

To further explore the relationship between *Lc–Pl* couplet medicines in the neural activity ligand–receptor interaction pathway, we mapped the relevant targets to this pathway, as shown in Figure 8. A total of 22 targets were involved in this pathway. As seen in the figure, the medicines mainly act on the neural activity ligand–receptor interaction pathway by regulating the expression of nerve-related targets such as acetylcholine receptors (enriched genes CHRM1, CHRM2, CHRM3, CHRM4, CHRM5, and CHRNA7), adrenergic receptors (enriched genes ADRA1D, ADRA1B, ADRA1A, ADRA2A, ADRA2B, ADRA2C, ADRB1, and ADRB2), dopamine receptors (enriched gene DRD1), 5-HT receptors (enriched gene HTR2A), opioid receptors (enriched genes OPRD1 and OPRM1), gamma-aminobutyric acid (GABA) receptors (enriched genes GABRA1 and GABRA3), glutamate receptors (enriched gene GRIA2), and capsaicin receptors (enriched gene TRPV1), thus playing a role in the treatment of migraines.

## Discussion

Based on a network pharmacology approach, we collected a total of 28 active ingredients of *Lc–Pl* couplet medicines, including ligustrazine, senkyunolide E, piperlonguminine, and piperine, and 11 important ingredients. Some of these, including the calcitonin gene-related peptide receptor antagonist folic acid (Lars, 2008; Oosterhout et al., 2010; Yi et al., 2021) and the TRPV1 inhibitor ligustrazine (Bai et al., 2016), have been documented in the literature as having treatment effects for migraine. We collected 11 important targets of *Lc–Pl* couplet medicines for migraine, including PTGS2, ADRB2, and SCN5A, and six core targets, including HSPA5, EEF1A1, and FUS. We identified 15 involved biological processes, including blood circulation, circulatory system processes and synaptic signal transmission, and 20 important signal pathways, including the neural activity ligand–receptor interaction pathway, the calcium signalling pathway and the cGMP PKG signal pathway.

In terms of the direct action targets of the chemical components of *Lc–Pl* couplet medicines on migraine, the two targets of cyclooxygenase 2 (PTGS2/COX2) and β2-adrenergic receptor (ADRB2) were correlated with the highest numbers of chemical components (18 and 15, respectively), indicating that these targets are closely related to *Lc–Pl* couplet medicines.

The existing research on migraine suggests that neurogenic inflammation is the key link in its onset (Dalessio, 1976) and that pro-inflammatory mediators play both peripheral and central roles in its pathophysiological mechanism (Ince et al., 2015). The PTGS2 gene is a key enzyme for the synthesis of prostaglandin, an inflammatory mediator that research (Huang et al., 2014) has shown is one of the pain-causing factors of migraine. The chemical components of *Lc–Pl* couplet medicines include myricetone, senkyunolide A, piperine, and piplartin, which bind to targets such as PTGS2 and exert anti-inflammatory effects through multiple pathways, including the tumour necrosis factor, interleukin-17 and nuclear factor kappa B signalling pathways.

The ADRB2 gene has a series of biological effects due to its regulation of the level of the second messenger cAMP in the cell. It is widely expressed in a variety of tissue cells (Litonjua et al., 2010) and plays an important role in the occurrence of migraine (Bhakta et al., 2021). Chemical components such as senkyunolide E, ferulic acid, piperlonguminine, and pipernonaline bind to targets including ADRB2 and ADRA2B and play the role of dilating cerebral vessels through multiple pathways (neural activity ligand–receptor interaction pathway, calcium signalling pathway, cGMP PKG signalling pathway, cAMP signalling pathway, etc.).

Calcitonin gene-related peptide (CGRP) is a marker of trigeminal microvascular activation and is widely believed to be an important factor in migraine attacks (Wattiez et al., 2020). A previous clinical study of a combination of three herbs, Radix Angelicae dahuricae (Baizhi), Rhizome Ligustici (Chuanxiong), and Folium Camelliae Sinensis (green tea), for the treatment of migraine demonstrated a significant reduction in CGRP levels in the administered group compared to the control group (Li et al., 2011). The latest study demonstrated that the compound Danggui Chuangge granule could decrease the contents of CGRP in rat plasma (Song et al., 2019). Based on previous studies and clinical trials, an in-depth investigation of CGRP is necessary for the treatment of migraine.

From analysing the KEGG pathway, we found that *Lc–Pl* couplet medicines mainly treat migraines via the neural activity ligand–receptor interaction pathway, the calcium signalling pathway, the cGMP-PKG signalling pathway, pathways in cancer and the cAMP signalling pathway. Approximately one-third of the identified targets are involved in the neural activity ligand–receptor interaction pathway, including adrenergic receptors [ADRA2B (Ni et al., 2010) and ADRB2 (Bayerer et al., 2010)], dopamine receptors [DRD1 (Shepherd et al., 2010)], 5-HT receptors [HTR2A (Nyholt et al., 1996; Yücel et al., 2016)], opioid receptors [OPRM1 (Wu)], GABA receptors [GABRA1 (Milena et al., 2020) and GABRA3 (Plummer et al., 2011)], glutamate receptors [GRIA2 (Gasparini et al., 2014)] and capsaicin receptors [TRPV1 (Meents et al., 2010)]. These receptors play an important role in the processes involved in migraine attacks. Therefore, *Lc–Pl* couplet medicines may relieve the symptoms of migraine by regulating changes in the content of these receptors, thus achieving a therapeutic effect.

With the development of systems biology, researchers have discovered that disease is a pathogenic factor that invades the biological system, disrupts the balance of the biological network and leads to abnormal functioning of the organism. In this way, the disease can be regarded as a specific interference of the biological network (del sol et al., 2010), and drug development strategies have correspondingly undergone a gradual shift from single-target, specific designs to combined multi-target drugs (Lu et al., 2012) intended to adjust the entire biological system. However, system-based drug design methods face many challenges (Csermely et al., 2005; Hopkins, 2008b). According to the theory underlying traditional Chinese medicine, the use of either a single drug or a combination of multiple drugs leads to an adjustment of the biological environment and a gradual correction of the unbalanced network to achieve the aim of treating diseases (Gertsch, 2011; Shao and Bo, 2013); this view is following the design concept of the multi-target combination medicine.

This paper has discussed the mechanisms of migraine treatment by *Lc–Pl* couplet medicines. Our findings are broadly consistent with existing research results, which provide a degree of support for the treatment of migraine with lutongning granules, composed exclusively of *Ligusticum chuanxiong* and *Piper longum*. A limitation of this study is that it only discusses predictability based on the network pharmacology approach; therefore, further experimental research is needed to verify the relevant targets and signal pathways.

## Data Availability

The original contributions presented in the study are included in the article/supplementary material, further inquiries can be directed to the corresponding authors.
