# Erratum: Exploring the pharmacological action mechanism of *Ligusticum Chuanxiong-Piper Longum* couplet medicines on the treatment of migraine based on network pharmacology

**DOI:** 10.3389/fphar.2022.1089017

**Published:** 2022-11-16

**Authors:** Frontiers Production Office

**Affiliations:** Frontiers Media SA, Lausanne, Switzerland

**Keywords:** *Ligusticum chuanxiong Hort.-Piper longum* L., migraine, network pharmacology, mechanism, neuroactive ligand-receptor interaction pathway

Due to a production error, the corresponding authors were incorrectly listed as Fang Lu and Tianjiao Lu. The correct corresponding authors are Tianjiao Lu and Chunze Zheng.

The publisher apologizes for this mistake. The original version of this article has been updated.

